# Supplementary material for: Seroprevalence and risk factors for Q fever and Rift Valley fever in pastoralists and their livestock in Afar, Ethiopia: A One Health approach
Source: PLoS Negl Trop Dis. 2024 Aug 23;18(8):e0012392. doi: 10.1371/journal.pntd.0012392 (PMC11376510; doi:10.1371/journal.pntd.0012392)
Supplement: Attachment — Submitted filename: Seroprevalence of C burnetii and RVF in Ethiopia.docx [file pntd.0012392.s001.docx]

**Seroprevalence and risk factors for Q Fever and Rift Valley Fever in pastoralists and their livestock in Afar, Ethiopia: A One Health approach**

The paper on the “Seroprevalence and risk factors for Q Fever and Rift Valley Fever in pastoralists and their livestock in Afar, Ethiopia: A One Health approach” addressed an important topic as both Q fever and RVF are neglected zoonoses that impact heavily on the livelihood of people in endemic communities.

The authors need to significantly revise the presentation of the following sections in the manuscript:

Within the methods section, sample size calculation subsection, line 128 – 129: the authors state that, “*The sample size was calculated for a precision of 0.05, a confidence of 95% and estimated seroprevalence of 30% in the pastoralists”. However, t*he sample size estimation for the two diseases in this section is not clear. Readers would expect to see the assumptions and sample size estimation for each disease (Q-fever and RVF) separately, and how the decision was made for which sample size to use from the two calculations for humans and animals. A minimum of four sample size estimations for human Q-fever, human RVF, animal Q-fever, and animal RVF need to be stated, then authors should inform readers the basis for the choice of the selected sample size for the human and for all/each animal species.

Under the Methods section, statistical analysis subsection, line 165 – 166: says, “*Uni- and multi-variable logistic regression models with a random effect on village level to account for clustering were performed to identify factors….”.* Earlier on in Line 135 they stated; ".....*the livestock were selected to match the same households as the pastoralists and included.......".* The statement in line 135 implies a cluster was defined by the household. However, line 165 indicates instead village level clustering was taken into account. The authors need to clarify whether the clusters were considered to be households/homesteads OR villages and the analysis should take into account the definition of "clustering" within the design.

Discussion section: The discussion need to be re-written following the conventional schorlarly approach and in line with the PLOS NTD journal guidelines. Under the "manuscript organization" section of the PLOS NTD guidelines, the instructions state that the discussion "should start with a brief summary of the main findings". What has been presented in the first paragraph of the discussion is contrary to the journal guidelines. In addition, the flow of the entire discussion is not in line with scholarly writing. A structured discussion is a strange approach to presenting, comparing, contrasting and arguing out the finding of one's work. The subheading should be removed from the discussion and the discussion structured to ensure coherency with themes in the preceding paragraph connecting well with those in the next paragraph. The discussion should in addition clearly articulate what the strengths, limitations, and public health relevancy of the study in Ethiopia and other Q-fever and RVF endemic settings.

**Minor comments**

Abstract section Line 44 – 45: “…..*while cattle abortions were found to be associated with Rift Valley Fever seropositivity in livestock…”.*

This reporting does not take into consideration what the exposures and outcomes are. This statement would be most appropriate if this was a case-control study.

Abstract section, line 51: “*and rapid response plans to be developed*”.

This was not an outbreak investigation. Therefore, this statement in the conclusion is inappropriate, and should be deleted.

Introduction section, line 112 – 113: “*This study aimed to determine the seroprevalence of C. burnetii and RVF virus in humans and their livestock and identify associated risk factors in the Afar region of Northern Ethiopia.”*

The aim of the study should be "to determine the seroprevalence of C. burnetii and RVF virus **antibodies** in humans......."

Methods section, line 122 – 123 and throughout the paper. Concerning the use of the word “woredas”.

The paper is written in English. The word "woredas" is not an English term and neither a noun, rather a local, descriptive term for an administrative unit within Ethiopia. The use in this paper should either be with inverted commas, “….‘woredas’ (districts) within the Afar........" Or first use put in brackets as in, "........in seven districts (woredas) within the Afar........".

Methods section, statistical analysis subsection, line 167 and throughout the manuscript: *“…multivariate analysis…”*

Is this the right term the authors intended to use? Do you mean “multivariable analysis” as used in line 165?

Results section, line 178: “*Pastoralists came from five woredas….”. For clarity to an international audience, use ‘district’ for “woredas” throughout the manuscript.*

*Results section, line 185: “….(first trimester for large ruminants or first semester for small ruminants)…”*

There is unnecessary repetition, as "first trimester for small and large ruminants" would suffice. OR Should it be different trimesters for small and large ruminants?

Results section, line 192: “*Apparent seroprevalence in pastoralists and livestock*”.

Need to qualify the subheading. For example, should it be "Apparent seroprevalence of *C. burnetii* and RVF among pastoralists and livestock in Afar, Ethiopia"?

Results section, line 199 – 200: Should it be "......pastoralists and livestock in Afar, Ethiopia"?

Results section, line 205 – 206: *"Apparent seroprevalence...." and "..True seroprevalence…."* , of what? Need to be concise in writing.

Results section, line 216 – 279: The presentation of results from the univariable analyses is exaggerated. You are aware that results from the univariable model are always distorted by their unadjusted confounders, and where adjustiment for confounding is done, it is important to present the adjusted OR results alongside the unadjusted results either in the same Table or in two separate Tables. It is also better to write the text for the univariable and multivariable results under the same subheading for seroprevalence in pastoralists or livestoke for each disease, highlighting mainlly those that remain independently associated with seropositivity after adjustiment.

Results section, table 3: use of *“Ref*” under odds ratio. Change to 1.

Results section, table 3: “*Livestock ownership”.* Which one is the reference livestock group?

Results section, table 3: *“Shepherding of livestock by:”* Did you classify this variable based on gender or based on sex? What was the rationale for separation of children from men and women?

Results section, line 234: *“Multivariate…”.* Should this be "multivariable"?

Results section, line 236: *“The multivariate model….”* shouldn't this be "multivariable model"?

Results section, line 241: “*The univariate model remained the model of best fit for RVF virus seropositivity in pastoralists.*” This is not entirely true. What this means is that all the factors associated with RVF seropositivity at the univariable level were confounded by other "possibly unknown " factors, thus all did not remain independently associated with RVF seropositivity after adjustiment for the known factors at the multivariable level.

Results section, line 243: Should this be "Univariable"?

There is a bias introduced by reporting and emphasis on the results of the univariable analysis. It would be important for the authors to succintly write on the factors associated with seropositivity after the multivariable regression.

Results section, line 252: “….*did not have goats….*” Should this be "did not have abortion events in goats" rather than "did not have goats"?

Results section, Table 4: *“Age, Breeder”.* What is this variable in terms of age?

Results section, line 265: “*Multivariate analysis of risk factors associated with seropositivity in livestock”.*

Does this subheading refer to C. burnetii or RVF? You should also consider whether to use the term "multivariable" rather than "multivariate". The Tables showing the results of the multivariable analysis are missing in the main results section, yet these are the most important and rational to present.

Discussion section, line 295 – 296: “*(search date: 23rd April 2023)”,* should be included in the reference not here in text OR is unnecessary where a citation has been made*.*

Discussion section, line 303*:”…. keeping herds often separated by”.* Separated by what?

Discussion section, line 309 – 310: *“Similarly, a study in livestock from pastoral regions in South-Eastern Ethiopia found a similar seroprevalence in goats (54.2%), but higher in camels (90.0%) and cattle (31.6%)”.*

Correct the structure of the sentence. Starts; "Similarly...........in South-Eastern Ethiopia found a similar.........". The word "similar" used twice in the same sentence!

Discussion section, line 320: “*Fig 1. Map of Afar, North-Eastern Ethiopia. Study sites shown in grey.*”

This map should be included in the results section not the discussion!

Discussion section, line 333: “…*commonly consumed – 20% also consume soured milk.*”

The use of a hyphen in this sentence is not appropriate.

Discussion section, line 339 – 340: “*…and as with C. burnetii those aged ≥ 49 years had increased odds of seropositivity.*”

Discussing about "C. burnetii" once again when the theme is about “RVF”?

Discussion section, line 340 – 342: “*The study in the Somali region by Ibrahim et al. was the only other study of RVF seroprevalence in humans in Ethiopia identified, reporting a higher prevalence of 13.2%*”.

Correct the structure of the English in the sentence.

Discussion section, line 352: use of *“woreda”.*

Discussion section, line 360: Use of *"e.g"* in a sentence and more so in brackets not appropriate.

Discussion section, line 364: “…*frequently taken…”* what does this mean?

*Discussion section, line 372 – 373: “We saw a similar significant association with livestock from households that had a history of abortions in cattle having a 2.5-fold increase in odds of being seropositive for RVF.”*

Is this correct? Was this significant association observed after discounting the influence of other factors. Authors need to be cautious about presenting results of univariable analysis.

Discussion section, line 376 – 377: “*Our study indicates that RVF is a contributing agent of abortion in this species.”*

Check the consistence of this conclusion with the results of the study.

Discussion section, line 388: “…..*Kyrgyzstan with a larger scale of comparison than our present study showed…*”

The statement "*with a larger scale of comparison than our present study*" is not coherent within the sentence and entire paragraph.

Discussion section, line 389 – 390: “*Indicating that human-animal regression of zoonoses may be dependent on the scale of comparison.”*

Starting a sentence with "indicating that..." may not appropriate grammar, and "*human-animal regression of zoonoses may be dependent on the scale of comparison.*" clouds the meaning of the sentence further.

Conclusion section, line 429 – 430: “*Q Fever (25.0% humans, 34.3% livestock) and RVF (6.1% human, 3.9% livestock)*”, results stated once again in the discussion/conclusion?

Last page of the paper ie after page 33: Map Figure presented a second time. Is this part of the results, discussion or supplementary material?
