# Supplementary material for: Seroprevalence and risk factors for Q fever and Rift Valley fever in pastoralists and their livestock in Afar, Ethiopia: A One Health approach
Source: PLoS Negl Trop Dis. 2024 Aug 23;18(8):e0012392. doi: 10.1371/journal.pntd.0012392 (PMC11376510; doi:10.1371/journal.pntd.0012392)
Supplement: Attachment — Submitted filename: Line by line revisions.docx [file pntd.0012392.s002.docx]

**Line by line revisions for the manuscript entitled “Seroprevalence and risk factors for Q Fever and Rift Valley Fever in pastoralists and their livestock in Afar, Ethiopia: A One Health approach”**

- Line 30ff: This sentence starting with “However” is giving mixed messages and arguments unrelated with each other (it reads as if the dependence of mobile pastoralist and their livestock poses a high risk for outbreaks). I think what the sentence is meant to transmit is “Mobile pastoralists are widely present in Ethiopia and their livelihoods as well as own health depend highly on the health of their livestock. Presence of Q fever and Rift Valley fever, as well as potential outbreak of these two diseases, however, pose a high risk to human and animal health, while also affecting human livelihoods.” OR “Potential outbreaks of these two diseases together with the high dependency of mobile pastoralists and their livestock, pose a high risk for human and animal health.”. Consider re-writing.
- Line 37: it wouldn´t be fair to claim it as “high”, there is no comparison at this point, neither a threshold. Consider removing adjectives as high, low ect. You might use lower or higher were applicable, because there you can make a comparison based on your won finding and data or compared to other data.
- Line 58: consider adding “through losses in livestock products, such as decreased milk production, loss of offsprings and low weight gain.”
- Line 60ff: it is not the exposure you documented, but rather the sero-prevalence or in lay words the presence of the diseases, consider re-wording. For being able to talk about the exposure you would need to have data at hand on the environment as well. Exposure does not equal to sero-prevalence/presence.
- Line 148: for Q fever serology the place of testing is mentioned, yet not for RVFV, consider adding this or if it is the same laboratory mentioned at AHRI then combining the two sections.
- Line 157: correcting the apparent prevalence using the Rogan Gladen estimation is slightly outdated in my opinion. It would have been investment worth choosing other statistical methods such as applying a Bayesian framework to adjust for imperfect diagnostic test characteristics and accounting for clustering in the study design, which sounds like is the case for your data, as you go by region-woredas-household. This is actually a multistage-cluster sampling design, which means the sample size calculation should actually also consider the ICC (intra-cluster correlation coefficient). Consider reflecting on why the ICC wasn´t used and why the AP correction was done using the RG method only. As described later on line 165ff, clustering was accounted for in the regression models however. Check Kostoulas P, Giovannini A, Alba A, Comin A, Meletis E, Iannetti S, et al. Harmonisation Of Transmissible disease Interpretation in the EU (HOTLINE). EFSA Support Publ [Internet]. 2019 Jul 18 [cited 2020 Aug 12];16(7). Available from: <http://doi.wiley.com/10.2903/sp.efsa.2019.EN-1678> for further reads.
- Line 181: what is the definition of “of breeding age”? Consider adding here in brackets. This is dependent on the cultural and livestock production practices of countries.
- Line 181ff: here it is the first time you mention the “household questionnaire data” which I assume corresponds to the data concerning demographichs and symptoms and syndromes such as abortion, which are later used for the regression analysis. I strongly advise describing these “household questionnaire data” beforehand in the Material and Methods section to make it clear for the reader what data this concerns and how it was collected. Where the household members asked verbally and retrospectively? Were the symptoms or other data observed over a certain time period? What was the occurrence rate of a symptom, what was the denominator in time? X time abortion over Y time span. Please elaborate.
- Line 181ff. consider adding time as a denominator for all of the descriptively described data on the frequency of observed/reported symptoms and events (selling animals, buying animals, mobility ect.)
- Line 192ff: what is the reason for the apparent sero-prevalence to be described in this detail, while later on the true prevalence is calculated and presented as well? As the TP is a corrected, more accurate version of the reality, I would argument there is no need to share the AP to this detail. If at all, the just add in one table together with TP, to show the difference. But even this is not one of the objectives of the study (to show sero-prevalences at the hand of different statistical calculation methods. Strongly suggest to re-think and revise.
- Line 209ff: but this is only because a test sensitivity and specificity of 100% each was assumed.
- Line 212ff: I advise to reconsider reasons for comparing AP and TP, and consider presenting only TP. Especially for those cases were tested and published Se and Sp data are available, TP should always be the preferred data to be shown and worth of discussion.
- Line 216ff: while risk factors are mentioned in the introduction and later here in the results, no mentioning of it in the M&M section. Strongly advise to add some data on this in the M&M section. What are the risk factors considered, what type of data was it (nominal, ordinal, continuous ect) how were they collected. Namely list the risk factors taken in to account for the regression analysis so the readers know what was considered. Potentially consider adding reasoning why these were selected. How was the dependet variables handled, was it sero-positivity at individual level or sero-prevalence at household level or other?
- Line 232 ff: several non-optimal data handling in my opinion –
  - Just differentiating between abortion and no abortion (and most likely you excluded those households with no of the specific species) is not precise enough to draw conclusions for whether a statistical association can be done towards disease seropositivity. Within what time period is this abortion supposed to have taken place, if at all, to be considered relevant to the seropositivity?
  - Same for migration. Were the categories yes or no only? Quite broad.
  - At least for the risk factors “ livestock purchased”, “livestock sold” and “stillborn” there is at least time denominator of time set as the past 12 months.
  - For the variable “shepherding of livestock” – these answer levels can´t be mutually exclusive. Can you really argue that it is either the men or only the women or only the children doing this? I can imagine that at different times different household members are involved in this task. This doesn´t reflect the reality in my experience. Also, what is the age limit of the category “children”? does there after come the adult (men and women)?
- Line 249ff: also here, what is missing is defining your risk factors, such as age category in animals. What do you consider as young or old? A listing, naming and explaining the different levels (iE young versus old) for each risk factor is much needed. You can do this in the text as you go, but must be in the M&M section, not here in the results the first time to be read, or you can add a table.
- Line 262ff: table 4. Same issues of the risk factors here, as with the regression analysis of the human seropositivity in my opinion. No denominator for abortion, uclear within that time period these counts were recorded, and the differentiation of women/men/children within the factor “shepherding of livestock by” is not well separated.
- Line 292ff: consider adding comparison with other Sahelian countries such as Chad and Mauretania and compare with other mobile pastoral settings, not only with and within Ethiopia.
- Line 399: I don´t think a Q fever outbreak in the Netherlands is comparable with an outbreak within the Sahel region. The environmental conditions (wind and weather), the infrastructure and population density per m2 as well as individuals immunity are not comparable. Consider choosing an example more compatible with the geographical setting of your own study.
- Generall: some points on limitations of the study and analysis should be added to the discussion.
