# Supplementary material for: Seroprevalence and risk factors for Q fever and Rift Valley fever in pastoralists and their livestock in Afar, Ethiopia: A One Health approach
Source: PLoS Negl Trop Dis. 2024 Aug 23;18(8):e0012392. doi: 10.1371/journal.pntd.0012392 (PMC11376510; doi:10.1371/journal.pntd.0012392)
Supplement: Attachment — Submitted filename: Response to reviewers comments_v2.1.docx [file pntd.0012392.s003.docx]

We would like to thank the reviewers for their careful consideration of our manuscript. We have made a number of changes based on their suggestions and believe this has improved clarity of our study. Please find our responses to the line-by-line comments below, the line references in our responses refer to the “track change” version of the manuscript.

--------------------
Methods

Reviewer #3:

-Are the objectives of the study clearly articulated with a clear testable hypothesis stated?
-> objectives yes, hypothesis no.

The hypothesis has been added at line 125-127.

-Is the population clearly described and appropriate for the hypothesis being tested?
-> What is not comptelety clear is why a different number of household from each study site (the 5 sites) were sampled. Further comments within the point by point revisions document (attached word file).

This study made further use of samples collected for a study on *Brucella* spp., however, not all samples collected in the *Brucella* study had enough volume left for inclusion in the current study on Q fever and RVF. Details provided at line 134-142, 155-158.

-Is the sample size sufficient to ensure adequate power to address the hypothesis being tested?
-> sample size was calculated suing appripriate tools, however clustering of the data (region-study site-hoseholds- humans/livestock) should have been considered. (More on this in the attached point by point revision.)

The Brucella study for which the samples were originally collected was a cross-sectional study that used multistage cluster sampling proportional to size, reference provided at line 135.

Tschopp R, Gebregiorgis A, Tassachew Y, Andualem H, Osman M, Waqjira MW, et al. Integrated human-animal sero-surveillance of Brucellosis in the pastoral Afar and Somali regions of Ethiopia. PLOS Neglected Tropical Diseases. 2021;15(8):e0009593.

The current study however was limited to the samples with adequate volume remaining to conduct the serological testing for *C. burnetii* and RVFV antibodies. This limitation is discussed in the discussion at line 543-548.

--------------------
Results

Reviewer #3: Results

-Does the analysis presented match the analysis plan?
-> in the analysis plan more detailed info on the so called risk factors should have been provided.

The variables included in the logistic regression analysis are provided in Table 1, at line 197.

--------------------
Conclusions

Reviewer #3:
-Are the limitations of analysis clearly described?
-> No, this is missing and should be added.

Limitations of the study have been added at lines 543-556.

--------------------
Summary and General Comments

Reviewer #1:
Line 69 & throughout - use serial commas (i.e., should be “… humans, animals, and their environment …”)
Thank you, this has been checked and corrected throughout the manuscript.

Line 71 & throughout - Q fever (not Q fever)

The term has been adjusted throughout the manuscript.

Line 72 & throughout - Rift Valley fever (not Fever)

The term has been adjusted throughout the manuscript.

Line 73 - RVFV is also under-reported due to poor infrastructure, poor public education/engagement, and severe economic impact from OIE/WOAH trade bans.

The additional points have been added as suggested, at line 77-78.

Line 89 - clarify “animals”; redundant given rest of sentence?

This sentence has been adapted for clarity.

Line 90 - through mosquitoes and other hematophagous arthropods

This sentence has been adapted as suggested.

Line 91 - “RVF” should be “RVFV” — check throughout for appropriate use of RVF vs RVFV.

Thank you, this has been checked and corrected throughout the manuscript.

Line 93 - establish the use of RVFV throughout instead of “RVF virus”

The term has been adjusted throughout the manuscript.

Line 103 - should be “… migrating and searching for …”

This sentence has been adapted for clarity.

Line 104 & throughout - suggest use either mobile or nomadic, not both; or clarify the terms

The term “nomadic” has replaced “mobile” when referring to pastoralists throughout the manuscript. The term “mobile is now used only for displaced populations.

Line 101 & throughout - no need to capitalize compass directions

This has been adjusted throughout the manuscript.

Line 107-111 — very good; should link anticipated results from statement in line 112-113 with suggested action to accomplish statement in line 107-111.

Thank you, the expected outcome of the study have been added at line 127-129.

Line 122 - were should be was

This sentence has been adapted as suggested.

Line 123 - should be “… representing five of the seven woredas, I.e., Amibara, Awash, …”

This sentence has been adapted as suggested.

Line 127 - remove comma

This comma has been removed.

Line 161 - should be “manufacturer’s”

This apostrophe has been added.

Line 163 - should be “reported”

This term has been corrected as suggested.

Line 182 - “was” should be “were”

This term has been corrected as suggested.

Line 303 - separated by … what?

This sentence has been corrected.

Line 324 - should be “… aged ≥49 years with C. burnetii seropositivity.”

This sentence has been corrected as suggested.

Line 355 - Aedes should be italicized

This has been corrected as suggested.

Line 366 & throughout - be consistent with capitalization of Woredas

“Woreda” has been replaced with “district” throughout the manuscript.

Line 377 - change “is” to “could be”

This sentence has been corrected as suggested.

Line 392 - change “lower” to “shorter”

This sentence has been corrected as suggested.

Line 399 - no comma after “drought”

This sentence has been corrected as suggested.

Line 553 - missing authors on Ref #32

This has been updated.

--------------------
**Line by line revisions for the manuscript entitled “Seroprevalence and risk factors for Q Fever and Rift Valley Fever in pastoralists and their livestock in Afar, Ethiopia: A One Health approach”**

- Line 30ff: This sentence starting with “However” is giving mixed messages and arguments unrelated with each other (it reads as if the dependence of mobile pastoralist and their livestock poses a high risk for outbreaks). I think what the sentence is meant to transmit is “Mobile pastoralists are widely present in Ethiopia and their livelihoods as well as own health depend highly on the health of their livestock. Presence of Q fever and Rift Valley fever, as well as potential outbreak of these two diseases, however, pose a high risk to human and animal health, while also affecting human livelihoods.” OR “Potential outbreaks of these two diseases together with the high dependency of mobile pastoralists and their livestock, pose a high risk for human and animal health.”. Consider re-writing.

This sentence has been rephrased at line 30-32.

- Line 37: it wouldn´t be fair to claim it as “high”, there is no comparison at this point, neither a threshold. Consider removing adjectives as high, low ect. You might use lower or higher were applicable, because there you can make a comparison based on your won finding and data or compared to other data.

The word “high” has been removed.

- Line 58: consider adding “through losses in livestock products, such as decreased milk production, loss of offsprings and low weight gain.”

This sentence has been adapted as suggested.

- Line 60ff: it is not the exposure you documented, but rather the sero-prevalence or in lay words the presence of the diseases, consider re-wording. For being able to talk about the exposure you would need to have data at hand on the environment as well. Exposure does not equal to sero-prevalence/presence.

This sentence has been rephrased as suggested.

- Line 148: for Q fever serology the place of testing is mentioned, yet not for RVFV, consider adding this or if it is the same laboratory mentioned at AHRI then combining the two sections.

The section on serological testing has been combined as suggested.

- Line 157: correcting the apparent prevalence using the Rogan Gladen estimation is slightly outdated in my opinion. It would have been investment worth choosing other statistical methods such as applying a Bayesian framework to adjust for imperfect diagnostic test characteristics and accounting for clustering in the study design, which sounds like is the case for your data, as you go by region-woredas-household. This is actually a multistage-cluster sampling design, which means the sample size calculation should actually also consider the ICC (intra-cluster correlation coefficient). Consider reflecting on why the ICC wasn´t used and why the AP correction was done using the RG method only. As described later on line 165ff, clustering was accounted for in the regression models however. Check Kostoulas P, Giovannini A, Alba A, Comin A, Meletis E, Iannetti S, et al. Harmonisation Of Transmissible disease Interpretation in the EU (HOTLINE). EFSA Support Publ [Internet]. 2019 Jul 18 [cited 2020 Aug 12];16(7). Available from: <http://doi.wiley.com/10.2903/sp.efsa.2019.EN-1678> for further reads.

We discussed this comment with our statistician and he agrees that accounting for imperfect diagnostic tests and clustering at the same time would be an elegant solution to estimate the true prevalence. He also pointed out that the point estimate of the true prevalence will be very similar to the Rogan Gladen estimator if the clusters are of similar size, the priors for SE and SP are roughly symmetric and all model assumptions are fulfilled. What would change is the confidence interval around the estimate, as such we have not presented the CIs.

The village represents the "ecological unit", as animals in the same villages often mix together. As such, the village can be considered the cluster while clustering at household level can be ignored.

We completely agree with the reviewer that a sample size determination adjusting for clustering would have been a better choice. Unfortunately, there is nothing we can do now beyond discussing this limitation (line 548-551). However, it is important to note that the sample size was used to estimate the prevalence with a certain precision. If our calculation was too optimistic the confidence interval of the estimate is getting broader (as we see in the prevalence estimates close to 30%: the one half length of the CI is rather 7%pts instead of 5%pts), however, this does not change our conclusions.

- Line 181: what is the definition of “of breeding age”? Consider adding here in brackets. This is dependent on the cultural and livestock production practices of countries.

A description of the variables measured has been added in Table 1, at line 197.

- Line 181ff: here it is the first time you mention the “household questionnaire data” which I assume corresponds to the data concerning demographichs and symptoms and syndromes such as abortion, which are later used for the regression analysis. I strongly advise describing these “household questionnaire data” beforehand in the Material and Methods section to make it clear for the reader what data this concerns and how it was collected. Where the household members asked verbally and retrospectively? Were the symptoms or other data observed over a certain time period? What was the occurrence rate of a symptom, what was the denominator in time? X time abortion over Y time span. Please elaborate.

Additional information on the variables from the household data have been added in the methods section in table 1, at line 197. The samples and data were collected for another study on brucellosis, the reference to the brucellosis study which provides more detailed methodology of the original sampling has been highlighted at line 135.

- Line 181ff. consider adding time as a denominator for all of the descriptively described data on the frequency of observed/reported symptoms and events (selling animals, buying animals, mobility ect.)

The time denominator has been added as suggested in the methods section (Table 1) and results tables.

- Line 192ff: what is the reason for the apparent sero-prevalence to be described in this detail, while later on the true prevalence is calculated and presented as well? As the TP is a corrected, more accurate version of the reality, I would argument there is no need to share the AP to this detail. If at all, the just add in one table together with TP, to show the difference. But even this is not one of the objectives of the study (to show sero-prevalences at the hand of different statistical calculation methods. Strongly suggest to re-think and revise.

Thank you, the detail on AP/TP has been reduced and included in one table (table 2, line 240)

- Line 209ff: but this is only because a test sensitivity and specificity of 100% each was assumed.

Correct, as there was no independent evaluation of this test only the manufacturer’s sensitivity and specificity data was available. This has been added to the limitations of the study at lines 551-556.

- Line 212ff: I advise to reconsider reasons for comparing AP and TP, and consider presenting only TP. Especially for those cases were tested and published Se and Sp data are available, TP should always be the preferred data to be shown and worth of discussion.

Both the AP and TP are now presented together in table 2 at line 237. The estimated TP for Q Fever could not be calculated for the IFA due to the test result being based on a composite result of two antibodies. Also, the 100% specificity and sensitivity reported by the manufacture for the ELISA may not be the most relevant for use in our population, this is discussed further in the limitations at line 553-556.

- Line 216ff: while risk factors are mentioned in the introduction and later here in the results, no mentioning of it in the M&M section. Strongly advise to add some data on this in the M&M section. What are the risk factors considered, what type of data was it (nominal, ordinal, continuous ect) how were they collected. Namely list the risk factors taken in to account for the regression analysis so the readers know what was considered. Potentially consider adding reasoning why these were selected. How was the dependet variables handled, was it sero-positivity at individual level or sero-prevalence at household level or other?

A description of the variables measured has been added in Table 1, at line 197. An explanation for the included variables is provided at line 190-192. Seropositivity was considered only at the individual level (line 189) as not all household/herd members were tested.

- Line 232 ff: several non-optimal data handling in my opinion –
  - Just differentiating between abortion and no abortion (and most likely you excluded those households with no of the specific species) is not precise enough to draw conclusions for whether a statistical association can be done towards disease seropositivity. Within what time period is this abortion supposed to have taken place, if at all, to be considered relevant to the seropositivity?
  - Same for migration. Were the categories yes or no only? Quite broad.
  - At least for the risk factors “ livestock purchased”, “livestock sold” and “stillborn” there is at least time denominator of time set as the past 12 months.
  - For the variable “shepherding of livestock” – these answer levels can´t be mutually exclusive. Can you really argue that it is either the men or only the women or only the children doing this? I can imagine that at different times different household members are involved in this task. This doesn´t reflect the reality in my experience. Also, what is the age limit of the category “children”? does there after come the adult (men and women)?

A description of the variables measured has been added in Table 1, at line 197. The time denominator has been added for all relevant variables. The variables of shepherding of livestock by men, women and children are independent of each other, the presentation of these results has been adjusted for better clarity. In this region men will generally be more involved with caring for large ruminants while women and children care for the small ruminants, this will of differ depending on household and herd composition.

- Line 249ff: also here, what is missing is defining your risk factors, such as age category in animals. What do you consider as young or old? A listing, naming and explaining the different levels (iE young versus old) for each risk factor is much needed. You can do this in the text as you go, but must be in the M&M section, not here in the results the first time to be read, or you can add a table.

A description of the variables measured has been added in Table 1, at line 197.

- Line 262ff: table 4. Same issues of the risk factors here, as with the regression analysis of the human seropositivity in my opinion. No denominator for abortion, uclear within that time period these counts were recorded, and the differentiation of women/men/children within the factor “shepherding of livestock by” is not well separated.

Please refer to the comments above.

- Line 292ff: consider adding comparison with other Sahelian countries such as Chad and Mauretania and compare with other mobile pastoral settings, not only with and within Ethiopia.

Thank you for the suggestion, further comparison with other pastoralist communities has been added.

- Line 399: I don´t think a Q fever outbreak in the Netherlands is comparable with an outbreak within the Sahel region. The environmental conditions (wind and weather), the infrastructure and population density per m2 as well as individuals immunity are not comparable. Consider choosing an example more compatible with the geographical setting of your own study.

Thank you for your comment. The case presented from the Netherlands was selected to show the possible magnitude of an outbreak with a well-documented example. As we’ve discussed in our manuscript, these diseases are often not diagnosed/reported in low-resource settings, and the scale of outbreaks in these settings therefore may not be fully recognized.

- Generall: some points on limitations of the study and analysis should be added to the discussion.

The limitations have been added at line 543-556.

--------------------
**Seroprevalence and risk factors for Q Fever and Rift Valley Fever in pastoralists and their livestock in Afar, Ethiopia: A One Health approach**

The paper on the “Seroprevalence and risk factors for Q Fever and Rift Valley Fever in pastoralists and their livestock in Afar, Ethiopia: A One Health approach” addressed an important topic as both Q fever and RVF are neglected zoonoses that impact heavily on the livelihood of people in endemic communities.

The authors need to significantly revise the presentation of the following sections in the manuscript:

Within the methods section, sample size calculation subsection, line 128 – 129: the authors state that, “*The sample size was calculated for a precision of 0.05, a confidence of 95% and estimated seroprevalence of 30% in the pastoralists”. However, t*he sample size estimation for the two diseases in this section is not clear. Readers would expect to see the assumptions and sample size estimation for each disease (Q-fever and RVF) separately, and how the decision was made for which sample size to use from the two calculations for humans and animals. A minimum of four sample size estimations for human Q-fever, human RVF, animal Q-fever, and animal RVF need to be stated, then authors should inform readers the basis for the choice of the selected sample size for the human and for all/each animal species.

Further details on the sample size calculation are provided at lines 145-158. Limitations of the sample size for livestock are discussed at line 546-548.

Under the Methods section, statistical analysis subsection, line 165 – 166: says, “*Uni- and multi-variable logistic regression models with a random effect on village level to account for clustering were performed to identify factors….”.* Earlier on in Line 135 they stated; ".....*the livestock were selected to match the same households as the pastoralists and included.......".* The statement in line 135 implies a cluster was defined by the household. However, line 165 indicates instead village level clustering was taken into account. The authors need to clarify whether the clusters were considered to be households/homesteads OR villages and the analysis should take into account the definition of "clustering" within the design.

The Brucella study used a village-level cluster sampling design. Village level clustering was used for the regression analysis.

Our study only included a subset of those collected for the brucellosis study (selected from the samples that had enough volume remaining). Livestock samples were selected to match the same households as the included pastoralists. This was done so we could determine if there was a correlation between seropositivity of pastoralists and their livestock.

Discussion section: The discussion need to be re-written following the conventional schorlarly approach and in line with the PLOS NTD journal guidelines. Under the "manuscript organization" section of the PLOS NTD guidelines, the instructions state that the discussion "should start with a brief summary of the main findings". What has been presented in the first paragraph of the discussion is contrary to the journal guidelines. In addition, the flow of the entire discussion is not in line with scholarly writing. A structured discussion is a strange approach to presenting, comparing, contrasting and arguing out the finding of one's work. The subheading should be removed from the discussion and the discussion structured to ensure coherency with themes in the preceding paragraph connecting well with those in the next paragraph. The discussion should in addition clearly articulate what the strengths, limitations, and public health relevancy of the study in Ethiopia and other Q-fever and RVF endemic settings.

The discussion has been rearranged and subheadings removed as suggested. Limitations are discussed at line 543-556.

**Minor comments**

Abstract section Line 44 – 45: “…..*while cattle abortions were found to be associated with Rift Valley Fever seropositivity in livestock…”.*

This reporting does not take into consideration what the exposures and outcomes are. This statement would be most appropriate if this was a case-control study.

This sentence has been re-phrased at line 46-48.

Abstract section, line 51: “*and rapid response plans to be developed*”.

This was not an outbreak investigation. Therefore, this statement in the conclusion is inappropriate, and should be deleted.

This statement has been deleted.

Introduction section, line 112 – 113: “*This study aimed to determine the seroprevalence of C. burnetii and RVF virus in humans and their livestock and identify associated risk factors in the Afar region of Northern Ethiopia.”*

The aim of the study should be "to determine the seroprevalence of C. burnetii and RVF virus **antibodies** in humans......."

This line sentence has been corrected as suggested.

Methods section, line 122 – 123 and throughout the paper. Concerning the use of the word “woredas”.

The paper is written in English. The word "woredas" is not an English term and neither a noun, rather a local, descriptive term for an administrative unit within Ethiopia. The use in this paper should either be with inverted commas, “….‘woredas’ (districts) within the Afar........" Or first use put in brackets as in, "........in seven districts (woredas) within the Afar........".

The term “woreda” has been replaced with “district” throughout the manuscript.

Methods section, statistical analysis subsection, line 167 and throughout the manuscript: *“…multivariate analysis…”*

Is this the right term the authors intended to use? Do you mean “multivariable analysis” as used in line 165?

The term has been corrected to “multivariable” throughout the manuscript.

Results section, line 178: “*Pastoralists came from five woredas….”. For clarity to an international audience, use ‘district’ for “woredas” throughout the manuscript.*

The term “woreda” has been replaced with “district” throughout the manuscript.

*Results section, line 185: “….(first trimester for large ruminants or first semester for small ruminants)…”*

There is unnecessary repetition, as "first trimester for small and large ruminants" would suffice. OR Should it be different trimesters for small and large ruminants?

The early stage abortion periods differ for the large (first **trimester**) and small ruminants (first **semester**).

Results section, line 192: “*Apparent seroprevalence in pastoralists and livestock*”.

Need to qualify the subheading. For example, should it be "Apparent seroprevalence of *C. burnetii* and RVF among pastoralists and livestock in Afar, Ethiopia"?

This sentence has been corrected as suggested.

Results section, line 199 – 200: Should it be "......pastoralists and livestock in Afar, Ethiopia"?

This sentence has been corrected as suggested.

Results section, line 205 – 206: *"Apparent seroprevalence...." and "..True seroprevalence…."* , of what? Need to be concise in writing.

This sentence has been corrected at line 227.

Results section, line 216 – 279: The presentation of results from the univariable analyses is exaggerated. You are aware that results from the univariable model are always distorted by their unadjusted confounders, and where adjustiment for confounding is done, it is important to present the adjusted OR results alongside the unadjusted results either in the same Table or in two separate Tables. It is also better to write the text for the univariable and multivariable results under the same subheading for seroprevalence in pastoralists or livestoke for each disease, highlighting mainlly those that remain independently associated with seropositivity after adjustiment.

The results tables and text have been rearranged as suggested.

Results section, table 3: use of *“Ref*” under odds ratio. Change to 1.

This has been changed as suggested.

Results section, table 3: “*Livestock ownership”.* Which one is the reference livestock group?

The reference is households that do not own that species (e.g. camel ownership vs not owning camels).

Results section, table 3: *“Shepherding of livestock by:”* Did you classify this variable based on gender or based on sex? What was the rationale for separation of children from men and women?

This variable is based on sex. Children were separated as along with women, they generally care more for small ruminants while men generally tend to the large ruminants. Although this may depend on the individual household composition and livestock kept.

Results section, line 234: *“Multivariate…”.* Should this be "multivariable"?

The term has been corrected to “multivariable” throughout the manuscript.

Results section, line 236: *“The multivariate model….”* shouldn't this be "multivariable model"?

The term has been corrected to “multivariable” throughout the manuscript.

Results section, line 241: “*The univariate model remained the model of best fit for RVF virus seropositivity in pastoralists.*” This is not entirely true. What this means is that all the factors associated with RVF seropositivity at the univariable level were confounded by other "possibly unknown " factors, thus all did not remain independently associated with RVF seropositivity after adjustiment for the known factors at the multivariable level.

This sentence has been rephrased at line 293-292.

Results section, line 243: Should this be "Univariable"?

There is a bias introduced by reporting and emphasis on the results of the univariable analysis. It would be important for the authors to succintly write on the factors associated with seropositivity after the multivariable regression.

The term “univariable” has been corrected throughout the manuscript and presentation results rearranged as suggested.

Results section, line 252: “….*did not have goats….*” Should this be "did not have abortion events in goats" rather than "did not have goats"?

This sentence has been rephrased for clarity, at line 317.

Results section, Table 4: *“Age, Breeder”.* What is this variable in terms of age?

These details have been added in the methods section in Table 1, line 197.

Results section, line 265: “*Multivariate analysis of risk factors associated with seropositivity in livestock”.*

Does this subheading refer to C. burnetii or RVF? You should also consider whether to use the term "multivariable" rather than "multivariate". The Tables showing the results of the multivariable analysis are missing in the main results section, yet these are the most important and rational to present.

The term “multivariate” has been corrected to “multivariable” throughout the manuscript. The results of the multivariable analysis have been added to the results tables.

Discussion section, line 295 – 296: “*(search date: 23rd April 2023)”,* should be included in the reference not here in text OR is unnecessary where a citation has been made*.*

The search date has been removed from the discussion section.

Discussion section, line 303*:”…. keeping herds often separated by”.* Separated by what?

This sentence has been corrected. The herds are separated by species.

Discussion section, line 309 – 310: *“Similarly, a study in livestock from pastoral regions in South-Eastern Ethiopia found a similar seroprevalence in goats (54.2%), but higher in camels (90.0%) and cattle (31.6%)”.*

Correct the structure of the sentence. Starts; "Similarly...........in South-Eastern Ethiopia found a similar.........". The word "similar" used twice in the same sentence!

The sentence has been rephrased at line 407-408.

Discussion section, line 320: “*Fig 1. Map of Afar, North-Eastern Ethiopia. Study sites shown in grey.*”

This map should be included in the results section not the discussion!

The map has been moved to the results section.

Discussion section, line 333: “…*commonly consumed – 20% also consume soured milk.*”

The use of a hyphen in this sentence is not appropriate.

The sentence has been rephrased at line 399-401.

Discussion section, line 339 – 340: “*…and as with C. burnetii those aged ≥ 49 years had increased odds of seropositivity.*”

Discussing about "C. burnetii" once again when the theme is about “RVF”?

The discussion has been rearranged as suggested.

Discussion section, line 340 – 342: “*The study in the Somali region by Ibrahim et al. was the only other study of RVF seroprevalence in humans in Ethiopia identified, reporting a higher prevalence of 13.2%*”.

Correct the structure of the English in the sentence.

The sentence has been rephrased at line 447 – 450.

Discussion section, line 352: use of *“woreda”.*

The term “woreda” has been replaced with “district” throughout the manuscript.

Discussion section, line 360: Use of *"e.g"* in a sentence and more so in brackets not appropriate.

The sentence has been rephrased at line 482-483.

Discussion section, line 364: “…*frequently taken…”* what does this mean?

The sentence has been rephrased at line 487.

*Discussion section, line 372 – 373: “We saw a similar significant association with livestock from households that had a history of abortions in cattle having a 2.5-fold increase in odds of being seropositive for RVF.”*

Is this correct? Was this significant association observed after discounting the influence of other factors. Authors need to be cautious about presenting results of univariable analysis.

Thank you, we have tempered this statement accordingly at line 456-459.

Discussion section, line 376 – 377: “*Our study indicates that RVF is a contributing agent of abortion in this species.”* Check the consistence of this conclusion with the results of the study.

This sentence has been rephrased at line 459-460.

Discussion section, line 388: “…..*Kyrgyzstan with a larger scale of comparison than our present study showed…*”

The statement "*with a larger scale of comparison than our present study*" is not coherent within the sentence and entire paragraph.

The sentence has been rephrased at line 510-511.

Discussion section, line 389 – 390: “*Indicating that human-animal regression of zoonoses may be dependent on the scale of comparison.”*

Starting a sentence with "indicating that..." may not appropriate grammar, and "*human-animal regression of zoonoses may be dependent on the scale of comparison.*" clouds the meaning of the sentence further.

The sentence has been rephrased at line 514-516.

Conclusion section, line 429 – 430: “*Q Fever (25.0% humans, 34.3% livestock) and RVF (6.1% human, 3.9% livestock)*”, results stated once again in the discussion/conclusion?

The results have been removed from the discussion section.

Last page of the paper ie after page 33: Map Figure presented a second time. Is this part of the results, discussion or supplementary material?

PLOS Neglected requires figures to also be uploaded as separate files but may have been combined in the PDF proof received by the reviewer?
