# Supplementary material for: Seroprevalence and risk factors for Q fever and Rift Valley fever in pastoralists and their livestock in Afar, Ethiopia: A One Health approach
Source: PLoS Negl Trop Dis. 2024 Aug 23;18(8):e0012392. doi: 10.1371/journal.pntd.0012392 (PMC11376510; doi:10.1371/journal.pntd.0012392)
Supplement: Attachment — Submitted filename: Q fever and RVF seroprevalence in Ethiopia.docx [file pntd.0012392.s004.docx]

**Seroprevalence and risk factors for Q fever and Rift Valley fever in pastoralists and their livestock in Afar, Ethiopia: A One Health approach**

The authors addressed most of the comments in their submission, and have significantly improved their manuscript, however, I have more concerns that need their attention.

Line 59: delete “potentially”.

Line 60: replace “burden” with “loss”.

Line 61 – 62 “through losses in livestock products, such as decreased milk production, loss of offspring and low weight gain”. Added text not necessary. Delete.

Line 69 – 70: “and the development of rapid response plans for potential outbreaks “. This conclusion is not appropriate to the results presented. This statement would be relevant if this was an outbreak investigation.

Line 78: “and severe economic impact from trade bans”. This highlighted statement does not fit with the others mentioned in the sentence. It fits to be a reason why reporting should be done, not why the disease is “under-diagnosed and under-reported”. Therefore, delete this statement from the sentence.

Line 87: “causing”. Replace with “to complications such as endocarditis, …….”.

This is because the sentence begins, “Chronic Q fever evolves from……” therefore should be followed with “to…..”

In addition, you need to specify whether you are referring to infection in humans or animals. I believe in this sentence you are referring to Q fever in humans, since the last sentence of this paragraph refers to animals. Therefore start the sentence as “Chronic Q fever in humans evolves from……..”

Line 112: “to changes to environment”. Just say, “to environmental changes such as…….”

Line 113 – 114: “in order to search for grazing areas”. Just say, “in search of grazing areas and water for their livestock.”

Line 152: “highest”. I think we calculate the “minimum” sample size, not the maximum.

Line 179 – 182: “For *C. burnetii*, apparent seroprevalence was calculated based on composite results of IgG Phase I and/or Phase II positivity. The estimated true prevalences (eTP) were determined by the Rogan–Gladen calculation based on the apparent prevalence (AP) and the sensitivities (Se) and specificities (Sp) of the diagnostic tests (1): eTP = (AP + Sp—1)/(Se + Sp—1).”.

Presentation of “apparent seroprevalence” and “estimated true prevalence”. The estimated true prevalence calculated from the apparent seroprevalence. The authors are presenting unnecessary details inform of two prevalence outputs. The main outcome (presence of Q fever or RVF antibodies) were obtained objectively using laboratory tests and not through subjective questioning. The presence of antibodies is a “hard outcome”, that requires no further conversion or serrogacy in this case. Since the data was analysed in STATA, the authors could directly calculate the seroprevalence with the syntax incorporating the variance-covariance matrix of the estimators.

At some point in the paper you state a failure to calculate the eTP due to the AP estimate from two antibody tests. This wouldn’t be a challenge if you defined your outcome clearly that “seropositivity is defined as testing positive to either …assay kit or …assay kit because the aim of the paper/work is not to compare AP and eTP.

Therefore, a single prevalence estimate should suffice. Presenting the AP and eTP for Q fever and RVF in humans and each of the livestock species adds only confusion, yet you would want your paper to be understood clearly by the scientific audience.

Line 193: “tested”. Should be “selected”

Line 197: “**Table 1. Variables included logistic regression analysis.**”. This table is not necessary as the same variables are indicated in the results tables. Please delete.

Line 207:" from 249 households”. Since you informed us that clustering was incorporated in the regression model at the village level, we expect to see the number of village clusters indicated as well.

Line 214: ‘ for 239 households. Livestock abortions were reported by 32.6% (78/239) of households”. You also need to tell us about the village level cluster. i.e. in how many village clusters?

Line 217 – 221: ‘Additionally, 35.1% (84/239) of households reported stillbirths among their livestock. All households interviewed in this study reported the practice of animal afterbirth disposal by discarding it in the bush. Selling livestock in the past 12 months was reported by 67.8% of households, while only 22.2% reported purchasing of new animals. The majority of households (72.4%) reported migration with their livestock. “.

Please, wherever you have household numbers, it is important to indicate the number of clusters to which they belong for clarity and transparency since the analysis incorporated the “village cluster” as the random intercept in the model.

Line 225: ‘Adapted from (2)*”.* Did you obtain permission from the original authors to use this map? If yes, then this statement should be written as “Adapted with permission from ….et al [33].

Line 233 – 237: ‘Estimated true prevalence (eTP) could not be calculated for *C. burnetii* in pastoralists due to the AP being based on a composite result of two antibodies. There was no difference in the AP and eTP for *C. burnetii* for livestock. The eTP of RVFV was slightly higher than the AP in both pastoralists (7.2%) and livestock (4.6%).”.

This is exactly what I was referring to above. The aim of the paper is to communicate the potential burden of Q fever and RVF. This should be done without complexity. The comparison or separate presentation of AP and/or eTP is not relevant and does not add insight to the paper. And please note: the regression analysis is not based on the results of either AP or eTP or which antibody test was used for each disease, but rather to whoever was generally counted as positive!

Line 240: “**Table 2. Seroprevalence of Coxiella burnetii and Rift Valley fever virus in pastoralists and livestock in Afar, Ethiopia**”. See my comments above.

Line 269 – 281: Here is the point where you create a contradiction in your analysis. If your cluster was defined as a village, then you should be presenting “….pastoralists from villages…”. But I believe your cluster must be defined as household, because this is what you refer to in nearly the entire paper. If you used ‘village cluster’ as your random intercept in the model, you need to check this and use the appropriate cluster level.

Line 293 – 294: “The univariable model remained the model of best fit for RVFV seropositivity in pastoralists when adjusted for the known variables”. I think you should change the language and just say, “All other factors were not associated with RVF seropositivity, thus multivariable analysis was not done.”

Line 315 – 317: Households or villages?

Line 329: “reduced odds”. Should be “reduced odds ratios”. Change as appropriate in other sections of the manuscript

Line 343: ‘increase in odds’ and in other parts of the paper. Take note of appropriate terminology….”odds” or “odds ratios”.

Line 358 - 362: For each multivariable analysis model, did you explore the possibility of interaction between pairs independent covariables before concluding these were the factors that best suited the model?

Did you check for a possibility of collinearity for example for factors such as “abortion events in cattle” and “abortion periods”?

Line 583 – 593: “Studies on zoonotic diseases in the Horn of Africa often have not followed a One Health approach, but rather consider human and animal health individually (3). However, to adequately control zoonotic diseases in these pastoral regions, a transdisciplinary approach is needed, involving the pastoral communities, the animal and public health authorities along with the research community (4).

Afar is a major pastoral region of Ethiopia in consideration of livestock numbers and importance to the regional economy in addition to cross-border movements of both humans and livestock to neighbouring countries within the Horn of Africa (5). Following the recent conflict in the Tigray region of Ethiopia, Afar has also seen an influx of internally displaced people migrating to the region. A scoping review on zoonotic diseases in the Horn of Africa that included 2,055 studies found Q fever to be comparatively understudied across the region, and both Q fever and RVF to be understudied in Ethiopia with each investigated in only 1% of publications (3). “.

This addition distorts the conclusion and all that is added here appears or should go to the introduction.

The remaining text is sufficient and appropriate for the conclusion.
