# Supplementary material for: Seroprevalence and risk factors for Q fever and Rift Valley fever in pastoralists and their livestock in Afar, Ethiopia: A One Health approach
Source: PLoS Negl Trop Dis. 2024 Aug 23;18(8):e0012392. doi: 10.1371/journal.pntd.0012392 (PMC11376510; doi:10.1371/journal.pntd.0012392)
Supplement: Attachment — Submitted filename: Response to reviewers comments.docx [file pntd.0012392.s005.docx]

We would like to thank the reviewers for taking the time to carefully assess our manuscript. Please find the point-by-point responses below.

____________________________________________________________________________________________________________________________________________

**Reviewer #2:** Seroprevalence and risk factors for Q fever and Rift Valley fever in pastoralists and their livestock in Afar, Ethiopia: A One Health approach

The authors addressed most of the comments in their submission, and have significantly improved their manuscript, however, I have more concerns that need their attention.

Line 59: delete “potentially”.

This line has been corrected as suggested.

Line 60: replace “burden” with “loss”.

This line has been corrected as suggested.

Line 61 – 62 “through losses in livestock products, such as decreased milk production, loss of offspring and low weight gain”. Added text not necessary. Delete.

This line has been corrected as suggested.

Line 69 – 70: “and the development of rapid response plans for potential outbreaks “. This conclusion is not appropriate to the results presented. This statement would be relevant if this was an outbreak investigation.

This sentence has been adjusted based on the reviewers comment.

Line 78: “and severe economic impact from trade bans”. This highlighted statement does not fit with the others mentioned in the sentence. It fits to be a reason why reporting should be done, not why the disease is “under-diagnosed and under-reported”. Therefore, delete this statement from the sentence.

This line has been corrected as suggested.

Line 87: “causing”. Replace with “to complications such as endocarditis, …….”.

This is because the sentence begins, “Chronic Q fever evolves from……” therefore should be followed with “to…..”

In addition, you need to specify whether you are referring to infection in humans or animals. I believe in this sentence you are referring to Q fever in humans, since the last sentence of this paragraph refers to animals. Therefore start the sentence as “Chronic Q fever in humans evolves from……..”

This line has been corrected as suggested.

Line 112: “to changes to environment”. Just say, “to environmental changes such as…….”

This line has been corrected as suggested.

Line 113 – 114: “in order to search for grazing areas”. Just say, “in search of grazing areas and water for their livestock.”

This line has been corrected as suggested.

Line 152: “highest”. I think we calculate the “minimum” sample size, not the maximum.

Due to the limited prevalence data available for the region we selected the highest sample size to ensure our prevalence calculation would have suffient power. Also, so that we would have enough positive samples to perform the regression analysis.

Line 179 – 182: “For *C. burnetii*, apparent seroprevalence was calculated based on composite results of IgG Phase I and/or Phase II positivity. The estimated true prevalences (eTP) were determined by the Rogan–Gladen calculation based on the apparent prevalence (AP) and the sensitivities (Se) and specificities (Sp) of the diagnostic tests (1): eTP = (AP + Sp—1)/(Se + Sp—1).”.

Presentation of “apparent seroprevalence” and “estimated true prevalence”. The estimated true prevalence calculated from the apparent seroprevalence. The authors are presenting unnecessary details inform of two prevalence outputs. The main outcome (presence of Q fever or RVF antibodies) were obtained objectively using laboratory tests and not through subjective questioning. The presence of antibodies is a “hard outcome”, that requires no further conversion or serrogacy in this case. Since the data was analysed in STATA, the authors could directly calculate the seroprevalence with the syntax incorporating the variance-covariance matrix of the estimators.

At some point in the paper you state a failure to calculate the eTP due to the AP estimate from two antibody tests. This wouldn’t be a challenge if you defined your outcome clearly that “seropositivity is defined as testing positive to either …assay kit or …assay kit because the aim of the paper/work is not to compare AP and eTP.

Therefore, a single prevalence estimate should suffice. Presenting the AP and eTP for Q fever and RVF in humans and each of the livestock species adds only confusion, yet you would want your paper to be understood clearly by the scientific audience.

The estimated prevalence has been removed.

Line 193: “tested”. Should be “selected”

This line has been corrected as suggested.

Line 197: “**Table 1. Variables included logistic regression analysis.**”. This table is not necessary as the same variables are indicated in the results tables. Please delete.

Table 1 was requested by another reviewer.

Line 207:" from 249 households”. Since you informed us that clustering was incorporated in the regression model at the village level, we expect to see the number of village clusters indicated as well.

This information has been added.

Line 214: ‘ for 239 households. Livestock abortions were reported by 32.6% (78/239) of households”. You also need to tell us about the village level cluster. i.e. in how many village clusters?

This information has been added.

Line 217 – 221: ‘Additionally, 35.1% (84/239) of households reported stillbirths among their livestock. All households interviewed in this study reported the practice of animal afterbirth disposal by discarding it in the bush. Selling livestock in the past 12 months was reported by 67.8% of households, while only 22.2% reported purchasing of new animals. The majority of households (72.4%) reported migration with their livestock. “.Please, wherever you have household numbers, it is important to indicate the number of clusters to which they belong for clarity and transparency since the analysis incorporated the “village cluster” as the random intercept in the model.

This information has been added.

Line 225: ‘Adapted from (2)*”.* Did you obtain permission from the original authors to use this map? If yes, then this statement should be written as “Adapted with permission from ….et al [33].

The map was updated to one with a CC BY 4.0 licence.

Line 233 – 237: ‘Estimated true prevalence (eTP) could not be calculated for *C. burnetii* in pastoralists due to the AP being based on a composite result of two antibodies. There was no difference in the AP and eTP for *C. burnetii* for livestock. The eTP of RVFV was slightly higher than the AP in both pastoralists (7.2%) and livestock (4.6%).”.

This is exactly what I was referring to above. The aim of the paper is to communicate the potential burden of Q fever and RVF. This should be done without complexity. The comparison or separate presentation of AP and/or eTP is not relevant and does not add insight to the paper. And please note: the regression analysis is not based on the results of either AP or eTP or which antibody test was used for each disease, but rather to whoever was generally counted as positive!

The estimated prevalence has been removed.

Line 240: “**Table 2. Seroprevalence of Coxiella burnetii and Rift Valley fever virus in pastoralists and livestock in Afar, Ethiopia**”. See my comments above.

The estimated prevalence has been removed.

Line 269 – 281: Here is the point where you create a contradiction in your analysis. If your cluster was defined as a village, then you should be presenting “….pastoralists from villages…”. But I believe your cluster must be defined as household, because this is what you refer to in nearly the entire paper. If you used ‘village cluster’ as your random intercept in the model, you need to check this and use the appropriate cluster level.

The original Brucella study for which the samples were collected used a village-level cluster sampling design. Village level clustering was used for the regression analysis as the village represents the "ecological unit". Data collected at the household level through household questionnaires are presented in the manuscript as “household variables”.

Line 293 – 294: “The univariable model remained the model of best fit for RVFV seropositivity in pastoralists when adjusted for the known variables”. I think you should change the language and just say, “All other factors were not associated with RVF seropositivity, thus multivariable analysis was not done.”

This sentence has been adjusted as suggested.

Line 315 – 317: Households or villages?

Households is correct.

Line 329: “reduced odds”. Should be “reduced odds ratios”. Change as appropriate in other sections of the manuscript

This has been adjusted as suggested.

Line 343: ‘increase in odds’ and in other parts of the paper. Take note of appropriate terminology….”odds” or “odds ratios”.

This has been adjusted as suggested.

Line 358 - 362: For each multivariable analysis model, did you explore the possibility of interaction between pairs independent covariables before concluding these were the factors that best suited the model?

Did you check for a possibility of collinearity for example for factors such as “abortion events in cattle” and “abortion periods”?

Thank you. There was no collinearity was found between the factors included in the multivariable analyses using the Pearson correlation coefficient.

Line 583 – 593: “Studies on zoonotic diseases in the Horn of Africa often have not followed a One Health approach, but rather consider human and animal health individually (3). However, to adequately control zoonotic diseases in these pastoral regions, a transdisciplinary approach is needed, involving the pastoral communities, the animal and public health authorities along with the research community (4).

Afar is a major pastoral region of Ethiopia in consideration of livestock numbers and importance to the regional economy in addition to cross-border movements of both humans and livestock to neighbouring countries within the Horn of Africa (5). Following the recent conflict in the Tigray region of Ethiopia, Afar has also seen an influx of internally displaced people migrating to the region. A scoping review on zoonotic diseases in the Horn of Africa that included 2,055 studies found Q fever to be comparatively understudied across the region, and both Q fever and RVF to be understudied in Ethiopia with each investigated in only 1% of publications (3). “.

This addition distorts the conclusion and all that is added here appears or should go to the introduction.

The remaining text is sufficient and appropriate for the conclusion.

This information has been moved to the introduction.

____________________________________________________________________________________________________________________

**Reviewer #3:** Thank you to the authors for their accomplished efforts in revising their manuscript accoding to reviewer guidance givven. I blieve the mansucript has improved technically, scientifically and in readability. Thank you also fo providing detailed explanation on where amanemends weren´t possible authors hae agrumented otherwise. This helped to understand the justufucation for sthe study conducted.

Thank you.
